# Detection of Solid-Phase Explosives Using an Electroantennogram-Based Biohybrid Sensor with Active Sniffing

**DOI:** 10.1021/acs.analchem.5c07760

**Published:** 2026-02-19

**Authors:** Rachel Rubinstein, Neta Shvil, Yossi Yovel, Amir Ayali, Ben M. Maoz

**Affiliations:** a School of Zoology, 26745 Tel Aviv University, Tel Aviv 69978, Israel; b Sagol School of Neuroscience, 26745 Tel Aviv University, Tel Aviv 69978, Israel; c School of Biomedical Engineering, 26745 Tel Aviv University, Tel Aviv 69978, Israel; d School of Mechanical Engineering, Tel Aviv University, Tel Aviv 69978, Israel; e The Center for Nanoscience and Nanotechnology, 26745 Tel Aviv University, Tel Aviv 69978, Israel; f Drimmer-Fischler Family Stem Cell Core Laboratory for Regenerative Medicine, 26745 Tel Aviv University, Tel Aviv 69978, Israel

## Abstract

Effective detection of hazardous compounds such as explosives is a critical objective in the fields of security and environmental monitoring. However, these materials, especially in their solid phase, present considerable analytical challenges due to their low vapor pressure and limited volatility under ambient conditions. In this study, we present a biohybrid sensing system that integrates an electroantennogram (EAG) recording from the antenna of the desert locust (*Schistocerca gregaria*), a bioinspired active sniffing mechanism, and machine learning assisted classification. This system enables noncontact detection of low-volatility compounds, without the need for heating, solvent extraction, or chemical preprocessing. It can reliably detect explosives such as trinitrotoluene (TNT), hexogen (RDX), and gunpowder and can discriminate TNT and RDX from nonexplosive solid odorants. A detection threshold of 2.67 pg was achieved for solid-phase TNT, which matches or even goes below previously reported detection approaches. These findings highlight the potential of insect-based biohybrid sensors as practical, low-cost tools for real-world chemical sensing, with promising applications in hazardous material monitoring.

## Graphical abstract

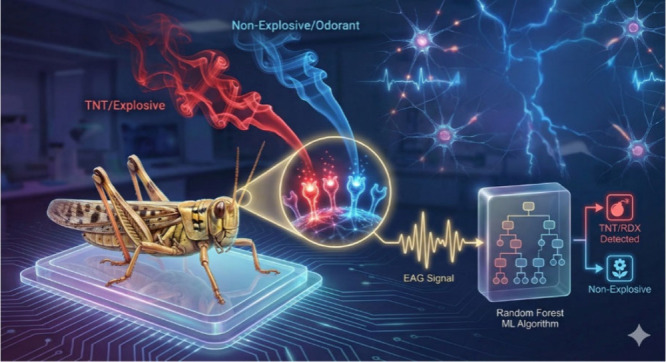

## Introduction

The demand for fast, low-cost, and sensitive detection of dangerous substances, such as drugs and explosives, is on the rise among law enforcement agencies and border control units worldwide.^1,2^ Detecting explosives, particularly in their solid form, poses a significant scientific and technological challenge due to their inherently low vapor pressures (VP).^2^ This low volatility means that only trace amounts of explosive molecules are present in the air, making vapor-phase detection extremely difficult. For instance, trinitrotoluene (TNT) has a VP of approximately 10^–8^ atm at room temperature, while hexogen (RDX) drops even lower, to around 10^–11^ atm^3^. In comparison, nonexplosive solids such as thymol (solid, ∼2.11 × 10^–5^ atm)^4^ and cinnamaldehyde (solid, ∼3.8 × 10^–5^ atm)^5^ are far more volatile. Furthermore, liquids exceed these values by several orders of magnitude—for example, acetone has a VP of approximately 0.3 atm^4^.

Traditional chemical and physical methods, such as chromatography and spectroscopy, can provide relatively accurate detection of explosives but often require expensive equipment, complex procedures, and lengthy processing times,^6^ and are less sensitive compared to many biological sensing systems.^7^ Another established approach for explosive detection is metal-oxide semiconductor (MOS) sensors, which offer high sensitivity and reliable responses, particularly when combined with machine learning tools.^8,9^ However, these devices consume substantial amounts of energy and exhibit limited selectivity, especially under variable environmental conditions such as humidity.^10^ Other electronic sensors, including e-noses and portable GCs, often suffer from limited sensitivity and selectivity and require samples to be collected manually, inserted into the device, and processed for analysis, a procedure that can be time-consuming.^11^ This approach precludes active, real-time environmental scanning and may expose operators to hazardous or even life-threatening conditions. Moreover, the detection mechanisms of many sensors are based on analyzing dissolved explosives, limiting their applicability for identifying solid-phase explosives in realistic field conditions.^12–15^

As a result of these constraints, Canines remain the gold standard for explosives detection due to their exceptional sensitivity and ability to discriminate odors in complex environments.^2,16^ Yet, their performance can be significantly affected by environmental factors like humidity and temperature, and often shows high variability that largely depends on their handler and motivation.^17,18^ Moreover, the cost, time, and effort required for their training are considerable.^19^ Other animals, including bees, African pouched rats, and dolphins, have also shown some promise.^2,16,20–22^ For example, bees trained via Pavlovian conditioning have been incorporated into devices like the Inscentinel Vasor, capable of detecting TNT at part-per-trillion levels.^2,23^ However, these systems necessitate working with live bees, which often suffer from limited operational windows (e.g., bees only function during daylight), present practical challenge, and need to be retrained frequently. Their conditioning loses effectiveness with repeated sessions, and they are highly sensitive to environmental variations.^24^

An alternative approach to training animals to detect volatile compounds is the integration of their sensory organs into biohybrid systems. Insects are particularly well-suited for this purpose as they are simple to rear and maintain, relatively inexpensive, pose fewer ethical challenges, and possess well-defined and accessible olfactory organs (their antennae).^25,26^

We have recently incorporated the desert locust antenna as a sensor into a biohybrid discriminator. This system used the electroantennogram (EAG) recording method with machine-learning classifiers to detect and discriminate more than eight different odors and their mixtures.^27^ With more than 50,000 olfactory receptor neurons (ORNs) per antenna, the desert locust exhibits a highly sensitive and broad olfactory detection system,^28–31^ making it an ideal candidate for the current study as well. The temporal response patterns of ORNs vary across different volatiles presented to the antenna. These electrophysiological signals can be used to train a machine learning algorithm for odor discrimination.

A crucial bioinspired aspect of our odorant detection system is the integration of active sampling mechanisms. Active sniffing not only allows operators to avoid direct contact with the hazardous material, but also significantly enhances vapor capture and sensor sensitivity.^32^ The concept of bioinspired active sniffing has recently been shown to improve detection thresholds by over an order of magnitude.^23^ Similar to the nonactive sensing methods, the most effective technology combining active sampling with solid-phase explosive detection to date is portable gas chromatography–mass spectrometry (GC-MS). However, this system demands substantial calibration, operator training, and expert-level data interpretation, and, as already mentioned, is less sensitive than its biological counterparts.^33–35^

In this paper, we present a biohybrid sensor that utilizes electroantennogram (EAG) recording of a locust antenna, a bioinspired active sniffing mechanism, and machine-learning tools, to detect and discriminate explosives in their solid form and at room temperature. This system is easy to operate and requires no further calibration after the initial training phase. It capitalizes on the high sensitivity of insect olfactory systems enhanced through controlled airflow and offers simpler, more intuitive interpretation of results compared to conventional systems. We demonstrate the system’s ability to detect low-volatility solids and explosives such as TNT, RDX, and gunpowder at various concentrations, showing characteristic dose–response behavior, without requiring dissolution or direct contact, offering a promising step toward robust, field-deployable explosive detection technologies.

## Results

### Detection and Discrimination of Solid Odorants

We first sought to determine whether the locust antenna could detect solid compounds, i.e., respond to their emitted volatile compounds. Experiments were performed using a custom-designed active sniffing apparatus, which enabled controlled odor delivery and electrophysiological recording (Figure 1). To ensure reliable measurements and to consider the decline in antennal activity following excision, all experiments were conducted within 30 min of antenna removal. During this time, antennal sensitivity remained relatively high, declining by only 28.5% (Figure SI1A & B).

**Figure 1:**
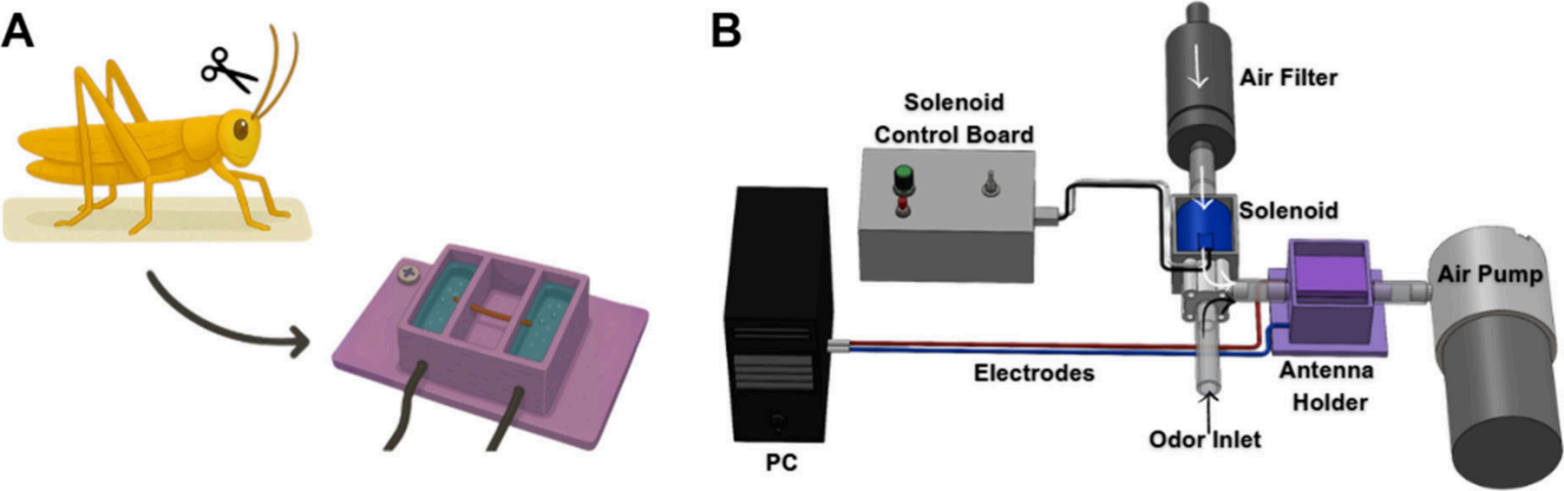
The recording and stimulus-delivery systems. (A) The antenna of the desert locust is removed and placed in a specialized antenna holder. The electrodes inserted into this holder record the differential voltage between the two tips of the antenna. (B) *Sniffer design:* When the solenoid is closed, filtered air flows through the antenna (white arrows), allowing it to acclimate to the airflow. When the solenoid opens, unfiltered ambient air replaces the filtered stream and reaches the antenna (black arrows).

Under these controlled experimental conditions, the locust antenna detected and generated distinct electrophysiological responses to each of the five tested solid odorants (Figure 2A,B, Table SI1). These responses were further analyzed using a random forest algorithm, which successfully discriminated between the odors with an accuracy of 80.9% (Figure 2C), significantly exceeding the expected random probability of 20%, as well as the randomized control accuracy of 18.43% (Figure 2D, Two-tailed binomial test: *p* < 0.0001). The algorithm proved to be robust to the high variability between repetitions of the same odor (Figure SI2A–E). The algorithm’s performance was further improved by subtracting the antenna’s mechanoreceptor response to airflow (i.e., the blank response to ambient air) during preprocessing (Figure SI2F). In this case, the same random forest algorithm achieved a discrimination accuracy of 82.7%, compared to only 22.7% in the shuffled-control condition (Figure SI2G,H, I, Two-tailed binomial test: *p* < 0.0001).

**Figure 2:**
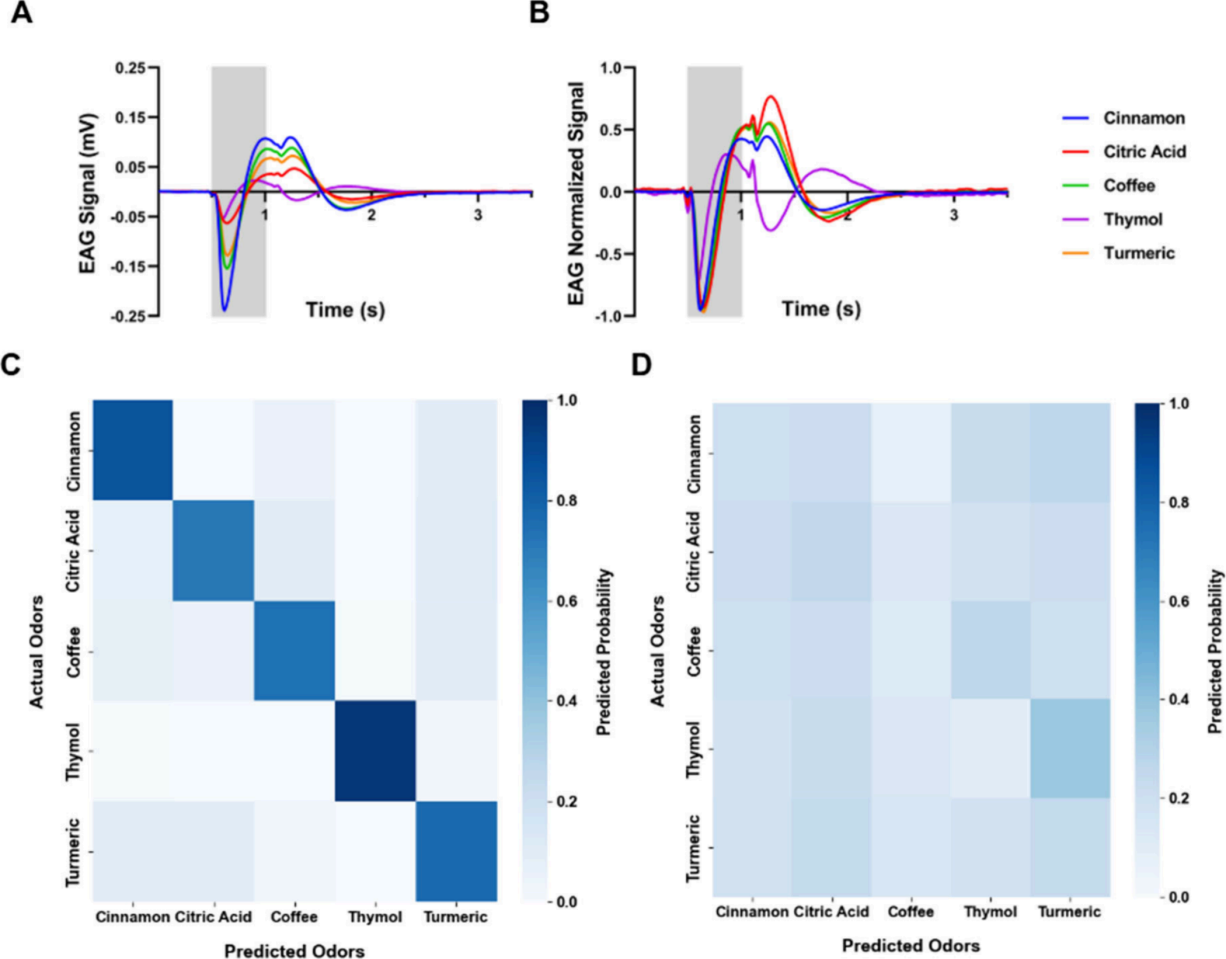
Solids discriminations. (A) Averaged and (B) averaged normalized EAG signals in response to 0.15 g of different solids representing the training data set. The stimulus duration is represented by the gray square. *N* = 30 antennae, 445 repetitions. (C) Confusion matrix for the discrimination training data set (normalized odorant responses as shown in panel B) using a random forest classifier, with an average accuracy of 80.9% over 445 samples. Two-tailed binomial test: *p* < 0.0001. (D) Control confusion matrix using randomized (shuffled) data with the random forest classifier, showing an average accuracy of 18.43% over 445 samples. Two-tailed binomial test: n.s.

### Detecting Solid Explosives

After evaluating the locust antenna’s responses to common solid compounds, we extended our analysis to explosives. Specifically, we examined the electrophysiological response of the antenna to three solid explosives in their native, unheated state: trinitrotoluene (TNT), gunpowder (GP), and hexogen (RDX). Despite their very low vapor pressures at room temperature (Table SI1), TNT, RDX, and GP elicited clear EAG responses that, as expected, were weaker than those to nonexplosive solids (Figure 3A). Heating the compounds to 50 °C appeared to enhance the antenna’s response (Figure 3B, example shown for TNT), but this approach is less applicable to real-world detection scenarios.

**Figure 3:**
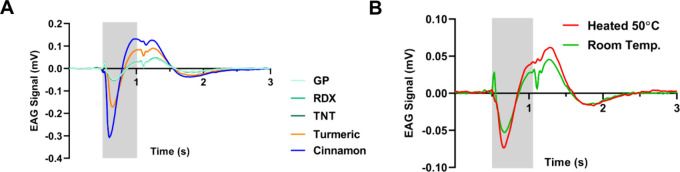
Explosive detection at room temperature. (A) Averaged EAG signals of 3 types of explosives (1 g) and two reference solids (0.15 g) at room temperature. *N* = 6 antennae, 6 repetitions for each odor. The stimulus duration is represented by the gray square. (B) Antenna response to 1 g of TNT at room temperature (RT, 25 °C) and at 50 °C. *N* = 8 and 6 antennae for heated and room temp, respectively. The stimulus duration is represented by the gray square.

The detection threshold of the system for each explosive was determined through dose–response measurements using varying masses of the odorant. As the mass of the tested explosive compound increased, the antennal response significantly intensified, primarily reflected in the amplitude of the negative peak. This phenomenon was observed for TNT and gunpowder but not for RDX. (Figure 4A,C,E, RM one-way ANOVA. TNT: *P* < 0.0001, GP: *P* < 0.0005, Kruskal–Wallis test for RDX: n.s.). A given amount was considered detectable if the absolute value of the negative trough of the signal (black arrow in Figure 4A) was greater than that observed in the blank air trials. For TNT, the lowest tested quantity (0.001 g) elicited a detectable response, whereas for GP and RDX, responses to amounts below 0.5 g did not surpass the detection threshold (Figure 4B,D,F).

**Figure 4:**
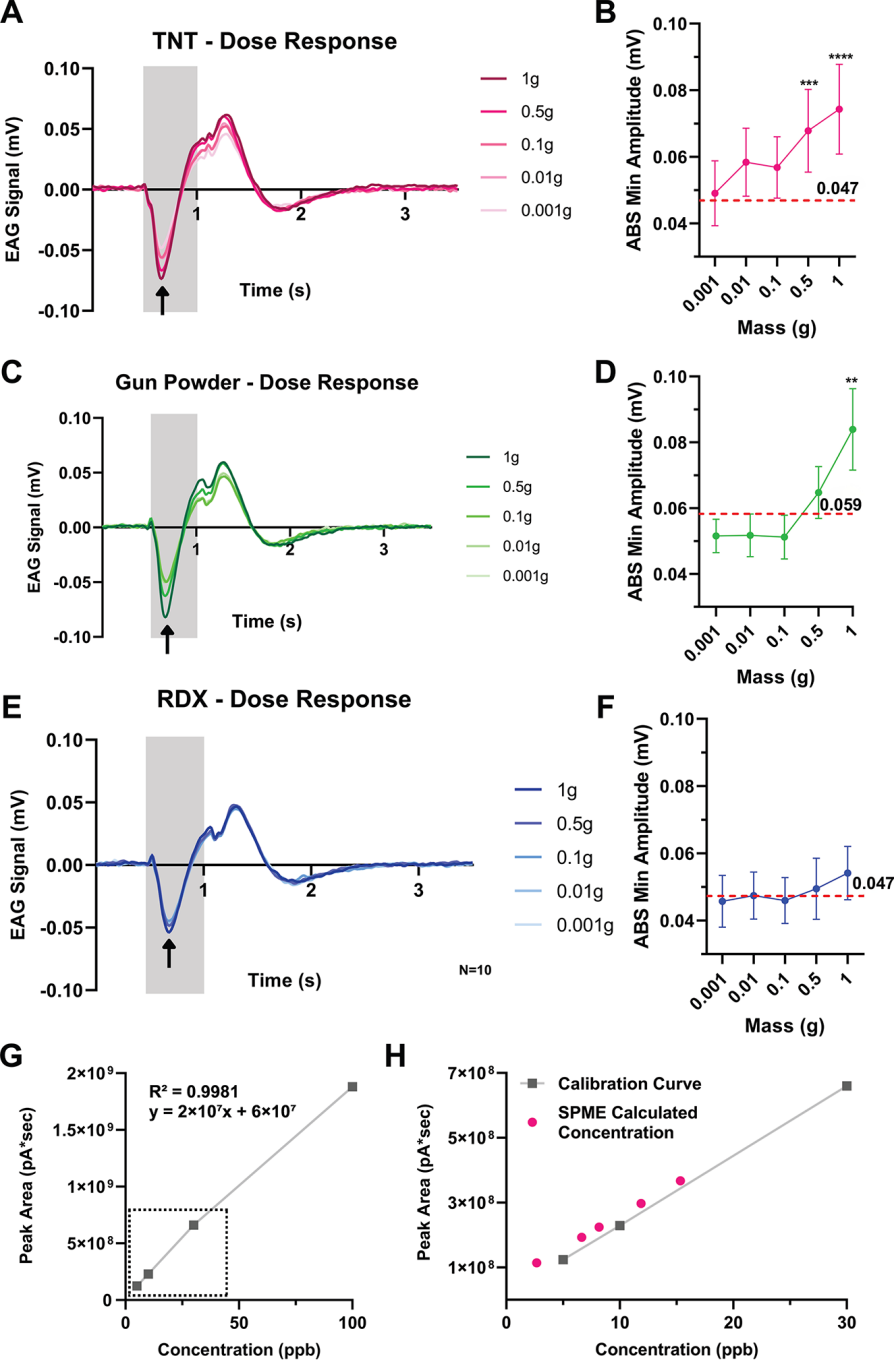
Explosives’ dose–response and threshold determination. (A, C, E) Response profiles of the locust antenna to different masses of TNT, gun powder, and RDX, respectively, ranging from 1 g to 0.001 g. *N* = 8, 9, and 10, respectively. The stimulus duration is represented by the gray square. (B, D, F) A dose–response graph (absolute value of the minimum signal’s amplitude per concentration) for TNT (data from A), gun powder (data from C) and RDX (data from E), respectively. The standard error of the mean (SEM) is depicted by vertical lines. RM one-way ANOVA for TNT: *P* < 0.0001 and GP: *P* < 0.0005, Kruskal–Wallis test for RDX: n.s. (G) Calibration curve for TNT, generated by measuring GC-ECD chromatographic peak area at concentrations of 5, 10, 30, and 100 ppb. (H) TNT concentrations absorbed by a SPME fiber, calculated using the linear equation of the calibration curve (from G). Results are shown on a zoomed-in section of the curve (dashed square in (G).

### Gas Chromatography-Electron Capture Detection (GC-ECD) for Determining Antennal TNT Detection Threshold

To estimate the amount of TNT delivered to the antenna during active sniffing, a second dose–response experiment was conducted using Solid Phase Microextraction (SPME) fiber instead of the antenna. A specialized antenna holder was constructed for the integration of the fiber with the sniffing system (Figure SI3A). Results were analyzed using Gas Chromatography with Electron Capture Detection (GC-ECD), where the area under curve (AUC) of the detected peak (Figure SI3B) was used to calculate the corresponding TNT concentrations based on the linear equation of a calibration curve’s trendline (R^2^ = 0.99, *y* = 2 × 10^7^ *x* + 6 × 10^7^, Figure 4G, Table SI2). Results show that the locust antenna was able to detect concentrations as low as 2.67 ppb, corresponding to 2.67 pg of TNT, marking the detection threshold of our system (Figure 4H). Based on the concentration–response relationship, the antennal limit of detection (LOD) and limit of quantification (LOQ) were estimated as 2.55 and 8.51 ppb, respectively (LOD = 3σ/S; LOQ = 10σ/S).

### Explosives Classification

Finally, we evaluated our system’s ability to detect TNT and RDX among other odors. We trained the above-mentioned Random Forest machine learning algorithm on five odor types: TNT, RDX, thymol, turmeric, and ambient air. The model distinguished the five odors with a high accuracy of 71.86%, significantly above both the expected random probability of 20% and the randomized control accuracy of 21.36% (Figure 5A,B; two-tailed binomial test: *p* < 0.0001 and n.s., respectively). Accuracy increased when comparing TNT alone to the other solids (86.02%, Figure SI4A; two-tailed binomial test: *p* < 0.0001) or RDX alone to the others (84.5%, Figure SI4B; two-tailed binomial test: *p* < 0.0001). This is likely due to the system’s difficulty in discriminating between TNT and RDX when both are present in the same classification task (53.62%, Figure SI4C; two-tailed binomial test: n.s.). This is likely due to their highly similar EAG signal profiles (Figure 3A). Furthermore, robust discrimination performance was maintained even when TNT was measured in the presence of a strong background odor (lemon), achieving 89.55% accuracy (Figure SI4D; two-tailed binomial test: *p* < 0.0001 and n.s., respectively), suggesting effective system performance under realistic environmental conditions. It is important to note that the classification relies primarily on the temporal dynamics of the EAG signal rather than absolute response amplitude.

**Figure 5:**
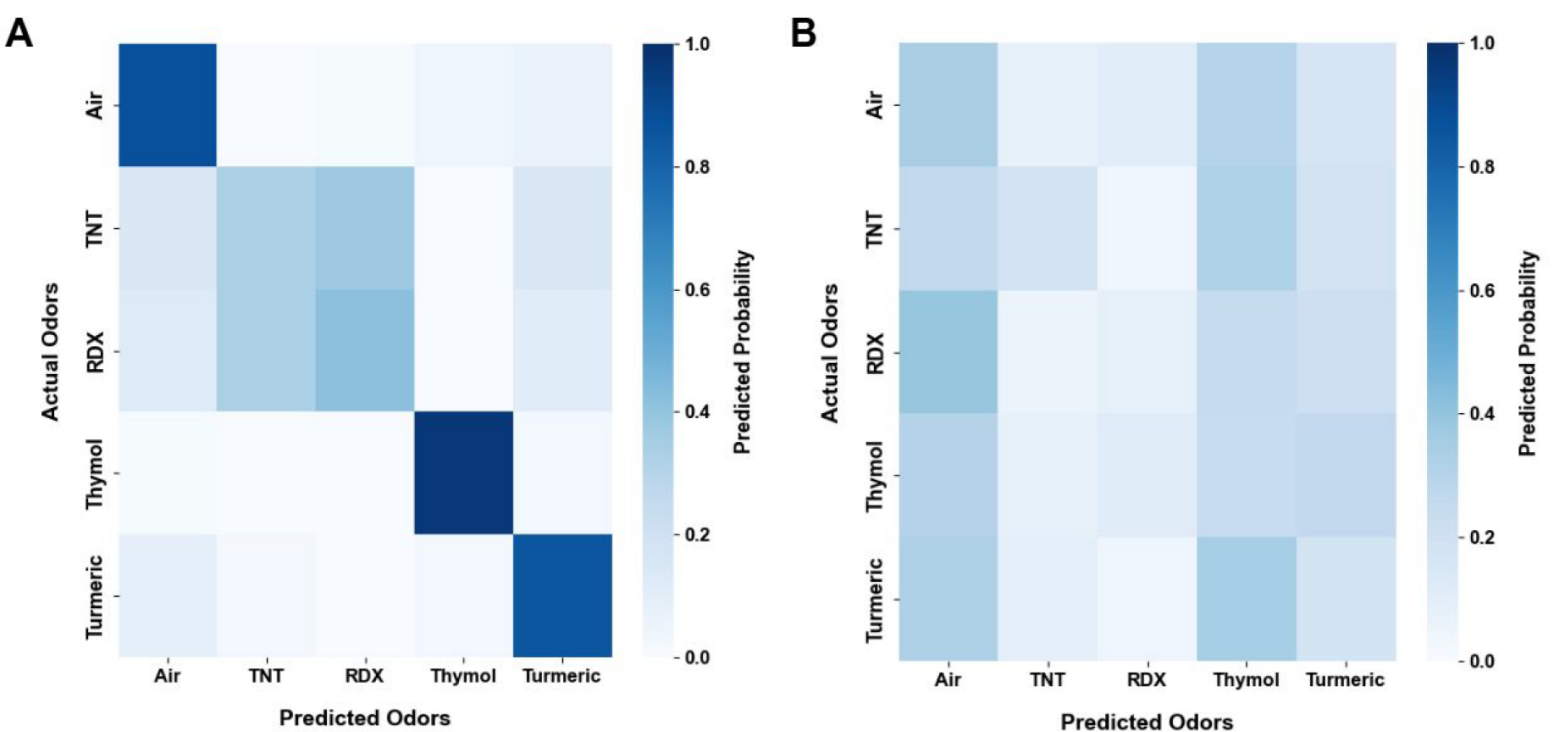
Explosives discrimination test. (A) Confusion matrix for two explosives, TNT and RDX vs two other solids and air using a random forest classifier, with an average accuracy of 71.86% over 398 samples. Two-tailed binomial test: *p* < 0.0001. (B) Control confusion matrix for panel A, with an average accuracy of 21.36% over 398 samples. Two-tailed binomial test: n.s.

## Discussion

This study presents a biohybrid sensing system that integrates the primary olfactory organ of the desert locust with electronic components to detect odors emitted from solid-phase compounds. Addressing the limitations of traditional detection systems and particularly their inefficiency in detecting low-volatility substances, this approach demonstrates an effective strategy for real-world chemical sensing, with promising applications in both environmental hazards monitoring and homeland security.

Biohybrid sensors that combine biological components offer a powerful alternative to conventional chemical sensors. Previously reported such sensors can be divided into mammalian-based (e.g., mouse olfactory bulb recordings, rat olfactory neurons^36,37^ ) and invertebrate-based approaches (e.g., locust antennal-lobe recordings^31^). While showing great promise, many of these systems rely on either exposing the biological sensors directly to explosive compounds dissolved in solution—often by injecting these solutions into the detection apparatus, or heating of the explosive compounds to artificially enhance vapor emission.^31,37^ These approaches do not reflect real-world scenarios, where such treatments are impractical or unsafe. Hence, in the current study we have employed a bioinspired active sensing device mimicking canines’ natural sniffing behavior. This device, recently developed and presented by Shvil et al. 2025,^32^ enhances vapor capture, enabling the highly sensitive detection of solid compounds. Furthermore, it is safe and practical for field conditions.

Dose–response experiments demonstrated that the locust antenna can detect trace quantities of explosive compounds, including TNT, RDX, and gunpowder. Using GC–ECD, the detection threshold for TNT was determined to be as low as 2.67 pg, sampled from a vial containing 0.001 g of solid TNT. Notably, the statistically estimated antennal limit of detection (LOD; 2.55 ppb) closely matches the experimentally observed threshold (2.67 ppb), indicating strong agreement between the calculated detection limit and the physiological response measured in the dose–response experiments. This detection threshold is at least 1 order of magnitude lower than those typically reported for other techniques, including fluorescence-based assays^14^ and advanced selective molecular sensors.^38^ While the analytical performance of conventional benchtop GC–ECD or GC–MS instruments is marginally superior to that of our system (reported LODs of 0.41 and 2.55 ppb, respectively),^39^ such measurements generally require direct handling and preprocessing of the explosive material, typically involving dilution. In contrast, our system achieves competitive sensitivity while enabling direct detection of low-volatility explosive compounds in their native solid phase, without heating, solvent extraction, or chemical processing. This demonstrates both high sensitivity and practical readiness for real-world deployment, where explosives are most commonly encountered in an unprocessed form.

As noted, substances’ vapor pressure strongly influences their detection. However, the antennal receptor affinity also plays a key role. Thus, a compound with lower vapor pressure may elicit a stronger signal than one with higher vapor pressure if its receptor affinity is greater. A well-known example is the moth’s ability to detect pheromones at extremely low concentrations, owing to the high affinity of its olfactory receptors.^40,41^ This phenomenon may also explain the differences in EAG response strength observed in the locust antenna. For example, turmeric—whose main volatile component, curcumin, has a vapor pressure of 4.08 × 10^–15^ atm—elicited a much higher EAG amplitude than thymol, which has a vapor pressure of 2.11 × 10^–5^ atm, or the tested explosives, whose vapor pressures range between 10^–9^ and 10^–12^ atm.^3,42–44^

A major advantage of the discrimination method based on EAG recordings is the high repeatability of the responses to the different odors. As all signals are normalized to the negative peak during preprocessing prior to algorithmic analysis, concentration- and intensity-dependent differences are eliminated, enabling the sensor to distinguish compounds even when their concentrations are unknown. The consistent activation patterns (temporal signal dynamics) generated by the antenna’s ORNs for each odor allow the trained discriminator to be applied continuously, without requiring retraining for each new locust or antenna prior to odor discrimination.

It must be noted that the locust antenna, being of biological origin, has a limited lifespan once removed from the insect. Although recent evidence suggests it can remain viable for up to 14 days under appropriate physiological conditions,^45^ its responsiveness decays exponentially over time. Since the antenna’s initial response to explosives is typically of low intensity, this decay must be carefully considered—particularly in dose–response experiments. To address this issue, all experiments reported herein were conducted within 15–30 min of antenna removal, when responsiveness is at its peak. Additionally, stimulus presentation order was randomized to minimize the effect of signal decay on the results.

At present, our system relies on a stationary amplifier, digital-to-analog converter, and computer for signal analysis. While this configuration provides highly sensitive and stable explosives detection, developing a portable version, as demonstrated in our recent study,^32^ could greatly enhance operational flexibility and enable on-site detection. Such a portable implementation would also allow the deployment of multiple sensing units, or even a coordinated swarm, where each unit incorporates a locust antenna as a biological sensing element. This approach would substantially increase the number of independent sensing events, thereby improving detection confidence and overall classification performance beyond the ∼ 80% accuracy achieved by a single sensor. Thus, the limitations of single-sensor accuracy are overcome, yielding an even more robust and scalable detection system, making it well suited for real-world security screening scenarios in which redundancy, rapid spatial coverage, and high detection confidence are essential.

Real-world environments contain background odors and odor mixtures that can introduce noise and interfere with signal detection.^46,47^ Our initial results demonstrated that our system can maintain discrimination of TNT even in the presence of a background odor, indicating robustness under more complex conditions. Future research could further train the system on odor mixtures or explosive odors presented alongside other disruptive background odors, complementing the pure-substance approach and enhancing performance in realistic, nonsterile environments.

To conclude, our findings present the biohybrid sensor based on insect antennae as a promising alternative to conventional detection technologies, particularly for field applications requiring rapid, sensitive, and noncontact sampling of solid-phase hazardous compounds. Such materials—including explosives, toxic chemicals, and other hazardous substances—pose significant challenges for existing sensors due to their low vapor pressure. The demonstrated ability of our biohybrid system to effectively detect trace amounts of solid compounds under ambient conditions highlights their potential for improved safety, environmental monitoring, and security screening. Moreover, its low production cost and operational simplicity make the system well-suited for deployment in a wide range of practical, real-world contexts.

## Materials and Methods

### Animals

All experiments were conducted using adult desert locusts (*Schistocerca gregaria*) of both sexes, from the breeding colony maintained at the School of Zoology, Tel Aviv University. The colony has been sustained over multiple generations in 60-L metal cages, each housing approximately 100–160 individuals. Environmental conditions were carefully regulated: room temperature was maintained at 30 °C and a 12-h light/dark cycle was used. To elevate daytime temperatures to 35–37 °C, additional radiant heat was supplied using 25 W incandescent bulbs. Locusts were provided with wheat seedlings and dry oats on a daily basis. Experiments were conducted in accordance with institutional regulations for invertebrate research, under which formal ethical approval is not required for insect use.

### Odorants and Explosives

The solid materials used in this work were citric acid (≥99.5%, ACS grade, Sigma-Aldrich), thymol (99–101%, Ph. Eur./BP/NF grade, Sigma-Aldrich), cinnamon and turmeric powder (Ta’am Vareach Spice company LTD, Petach Tikva, Israel), and coffee powder (Elite, Strauss Group). Solid standards trinitrotoluene (TNT), hexogen (RDX, 1,3,5-trinitro-1,3,5-triazinane) and gunpowder (potassium nitrate 75%, sulfur 10%, charcoal 15%) in small quantities were provided by the local law enforcement in accordance with the official guidelines for handling and storing explosives in the laboratory. The quantities used in the experiments were 1 g for explosives and 0.15 g for nonexplosive solids, unless stated otherwise.

### Electroantennogram Recordings

EAG recordings were conducted following the methodology established by Shvil et al.^27^ with minor adjustments. In brief, a locust antenna was removed, its tip trimmed and then secured in a custom 3D printed antenna holder such that both ends were immersed in conductive gel, while the middle section was left exposed to ambient air (Figure 1A). After a 5 min acclimation period to allow the antenna to adjust to airflow conditions, the experiment began. Electrical signals from the antenna olfactory receptor neurons, were captured using two silver electrodes (bare diameter: 0.025″; coated diameter: 0.030″; PFA-coated silver, A-M Systems, Sequim, WA) inserted into the two conductive gel wells. The signal was amplified by a factor of 100 with a four-channel differential AC amplifier (Model 1700, A-M Systems, Bellevue, WA, USA), bandpass-filtered (1 Hz-HPF/500 Hz-LPF), and digitized at a sampling rate of 100 Hz using a 2-channel signal acquisition interface IDAC -2 (SYNTECH, Hilversum, The Netherlands) to a PC using Auto-Spike software (SYNTECH, Germany, Figure 1B).

### The Sniffing System

We previously developed an active air sampling module that mimics biological sniffing by combining a solenoid-controlled pump with a custom-designed, airtight antenna holder featuring a two-lid system.^32^ To reduce tactile neuron responses, a custom carbon air filter was integrated into the system, providing a continuous flow of filtered air over the antenna. During each 0.5 s odor sampling “sniff” event, ambient air was drawn in and replaced the filtered stream, allowing for effective and controlled odor presentation (by a custom-built stimulus controller, manually triggered via button press, Figure 1B).

### Stimulus Exposure

Each odor was presented in a 1.5 mL Eppendorf tube kept at room temperature unless stated otherwise. For discrimination experiments, each stimulus was applied in three repetitions at 30s intervals, with 1 min intervals between different stimuli (Figures 2 and 5). In all other experiments, only a single repetition was applied. All stimulus exposures to a given antenna were completed within 30 min of antenna excision to minimize variability associated with postexcision decay of antennal activity (Figure SI1). In single-repetition experiments, stimulus exposure was completed within 15 min. Since the tested odorants were solid and could not be diluted by traditional serial dilution, before the dose–response experiment, we calibrated and compared two alternative approaches using a strongly reactive solid—coffee—as a test substance. In one method, different quantities of coffee powder were placed in identical vials (1 g, 0.1 g, 0.01 g, 0.001 g, and an “aroma” vial emptied but left unwashed), while in the other, equal amounts of substance were used but the duration of vial sealing was varied to modulate headspace concentration. The dose–response approach based on varying masses was ultimately selected, as it produced more distinguishable and higher-amplitude responses (Figure SI5). Accordingly, for the dose–response experiments with explosives, decreasing masses (1, 0.5, 0.1, 0.01, and 0.001 g) were placed in Eppendorf tubes and heated to 50 °C to increase vapor emission, with empty tubes serving as blank controls to assess detection thresholds. In all experiments, each tested antenna was exposed to all odors, with the odor order randomized across antennae to avoid order-related bias.

For the subset of TNT measurements conducted in the presence of a background odor, the experimental room was infused with a lemon scent by heating 1 mL of lemon oil (diluted 1:10 with mineral oil) on a candle burner for 5 min. The odor source was positioned 30 cm from the antenna holder.

### Gas Chromatography-Electron Capture Detection (GC-ECD)

TNT levels were quantified using Solid Phase Microextraction (SPME) fiber followed by thermal desorption into the GC-ECD inlet for analysis. A custom-made SPME holder was designed using SolidWorks (SolidWorks, Corporation, MA) and printed using a Raise 3D Pro2 Dual Extruder 3D Printer (Raise Technologies, Inc.) to ensure smooth integration with our system (Figure SI3A). To estimate the amount of TNT delivered during active sniffing, we used an SPME fiber exposed under experimental conditions identical to those used for antennal recordings, providing a standardized approximation of the delivered dose. While this SPME-based measurement does not aim to replicate the exact adsorption properties of the antennal surface, it yields a consistent and conservative estimate of the TNT presented under our experimental conditions. A 100 μm polydimethylsiloxane (PDMS) SPME fiber (24 Ga; Supelco PN: 57300-U, Sigma Israel) was exposed to odor stimulus under experimental conditions identical to those used in the original dose–response experiment: a range of 0.001–1g TNT, heated to 50 °C, in 1.5 mL Eppendorf tubes and half a second stimulus duration. Immediately after the sampling, the SPME fiber was desorbed at 250 degrees into the GC (6890N, Agilent) inlet at splitless mode. TNT was separated using RTX-vTNT2 Cap Column (PN 12999, Restek) and detected by micro-ECD detector (Agilent). TNT was identified from the chromatographic output by matching retention times to a TNT standard (5.64 min), allowing a deviation of ±0.01 min (Figure SI3B). The amounts of TNT adsorbed onto the fiber in the dose–response experiment were calculated using a linear equation obtained from a calibration curve constructed with TNT diluted in acetonitrile (HPLC grade, PN: 000120350200, BioLab) at concentrations of 5, 10, 30, and 100 ppb.

### Data Analysis

Recordings were analyzed as follows: median offset correction was applied individually to each measurement, and the corrected responses were then averaged across all antennae for each odor. Final values were plotted using GraphPad Prism (version 8.0.1). For TNT recordings obtained in the presence of the lemon background odor, the ambient air signal (i.e., the response to the background odor alone) was additionally subtracted to correct for background odor interference.

Data processing was performed using Python 3 (Python Software Foundation, Scotts Valley, CA) via a custom-built graphical user interface.^27^ This interface implemented all analytical steps mentioned above and enabled visualization, organization, and export of the results in formats compatible with Excel and Prism.

### Machine Learning Algorithm

Solids classification was performed using a random-forest classifier (n_estimators = 500, criterion = ’entropy’, max_depth = 8, max_features = ’auto’) implemented in Python 3 (Python Software Foundation, Scotts Valley, CA). The classifier input consisted of the first 250 samples of the normalized EAG time series, sampled at 100 Hz. During preprocessing, each trial was normalized such that the minimum value was scaled to – 1 (following offset correction; see Data Analysis). Data were split into training and testing sets using a leave-one-group-out cross-validation approach, in which each antenna was treated as a distinct group. In each fold, recordings from a single antenna served as the test set, while all other data were used for training (Figure SI2G). This approach ensured that data from the same antenna did not appear in both training and testing sets. Classification performance was evaluated using balanced accuracy.

### Statistics

To assess the significance of the classifier’s performance, we used a two-tailed binomial test, with chance defined based on the number of classes, and N representing the number of antennae tested. To test the antennal response significance in the dose–response experiments we used repeated measures one-way ANOVA for TNT and gunpowder and Kruskal–Wallis test for RDX (since RDX data did not pass normality test).

## Supplementary Material



## Notes

Table SI1 listing vapor pressures of various substances reported in the literature; Table SI2 listing TNT detection threshold (ppb) calculated based on GC-ECD; Figure SI1 showing antenna decay following excision; Figure SI2 showing solids discrimination and machine learning algorithm training; Figure SI3 showing GC-ECD setup and output overview; Figure SI4 showing explosives discrimination test; Figure SI5 showing dose–response procedure calibration (PDF)
