# Supplementary material for: Detection of Solid-Phase Explosives Using an Electroantennogram-Based Biohybrid Sensor with Active Sniffing
Source: Anal Chem. 2026 Feb 19;98(8):6295–303. doi: 10.1021/acs.analchem.5c07760 (PMC12961636; doi:10.1021/acs.analchem.5c07760)
Supplement: Supplementary file 1 [file ac5c07760_si_001.pdf]

Detection of Solid-Phase Explosives Using an Electroantennogram-Based Bio-Hybrid Sensor with Active Sniffing

Rachel Rubinstein[a#], Neta Shvil[b#], Yossi Yovel[a,b,d***], Amir Ayali[a,b**], Ben M. Maoz[b,c,e,f*].

a School of Zoology, Tel Aviv University, Tel Aviv 69978, Israel

b Sagol School of Neuroscience, Tel Aviv University, Tel Aviv 69978, Israel

c School of Biomedical Engineering, Tel Aviv University, Tel Aviv 69978, Israel

d School of Mechanical Engineering, Tel Aviv University, Tel Aviv 69978, Israel

e The Center for Nanoscience and Nanotechnology, Tel Aviv University, Tel Aviv 69978, Israel

f Drimmer-Fischler Family Stem Cell Core Laboratory for Regenerative Medicine, Tel Aviv University, Tel Aviv 69978, Israel

#These authors have contributed equally

* Corresponding author. Sagol School of Neuroscience, Tel Aviv University, Tel Aviv, Israel.

** Corresponding author. School of Zoology, Tel Aviv University, Tel Aviv, Israel.

*** Corresponding author. School of Zoology, Tel Aviv University, Tel Aviv, Israel.

E-mail addresses: yossiyovel@gmail.com (Y. Yossi), ayali@tauex.tau.ac.il (A. Amir), bmaoz@tauex.tau.ac.il (M.M. Ben).

Key Words: Insect, Antenna, Olfaction, Odorant, Chemical sensing, TNT, RDX

Supplementary Information

Table SI1: Vapor pressures of various substances reported in literature.

Table SI2: TNT detection threshold (ppb) calculated based on the GC-ECD calibration-curve's equation.

Figure SI1: Antenna Decay Following Excision.

Figure SI2: Solids Discrimination and Machine Learning Algorithm Training.

Figure SI3: GC-ECD Setup and Output Overview.

Figure SI4: Explosives Discrimination Test.

Figure SI5: Dose-Response Procedure Calibration.

Supplementary Tables

Substance	Vapor pressure (atm units, at 25°C)	Reference
**2-Furfurylthiol (Coffee)**	5.26×10^{-3}	Furan-2-ylmethanethiol | CAS#:98-02-2 | Chemsrc[1]
**Cinnamaldehyde (Cinnamon)**	3.80×10^{-5}	Cinnamaldehyde | C9H8O | CID 637511 - PubChem[2]
**Citric Acid**	2.18×10^{-11}	Hazardous Substances Data Bank (HSDB): 2000 - PubChem [3]
**Curcumin (Turmeric)**	4.08×10^{-15}	Curcumin | C21H20O6 | CID 969516 - PubChem[4]
**Thymol**	2.11×10^{-5}	Hazardous Substances Data Bank (HSDB) : 866 - PubChem[5]
**Hexogen (RDX)**	4.85×10^{-12}	Ewing et al.[6]
**Trinitrotoluene (TNT)**	9.15×10^{-9}	Ewing et al.[6]
**Sulfur (Gunpowder)**	5.19×10^{-9} at 30.4 °C	Sulfur | S | CID 5362487 - PubChem[7]

Table SI1: Vapor pressures of various substances reported in literature

Peak Area (pA/sec)x 10^8	Concentration Calculated Based on the Equation (ppb)
**3.67**	**15.35**
**2.97**	**11.87**
**2.24**	**8.18**
**1.93**	**6.65**
**1.14**	**2.68**

Table SI2: TNT detection threshold (ppb) calculated based on the GC-ECD calibration-curve's equation.

Supplementary Figures

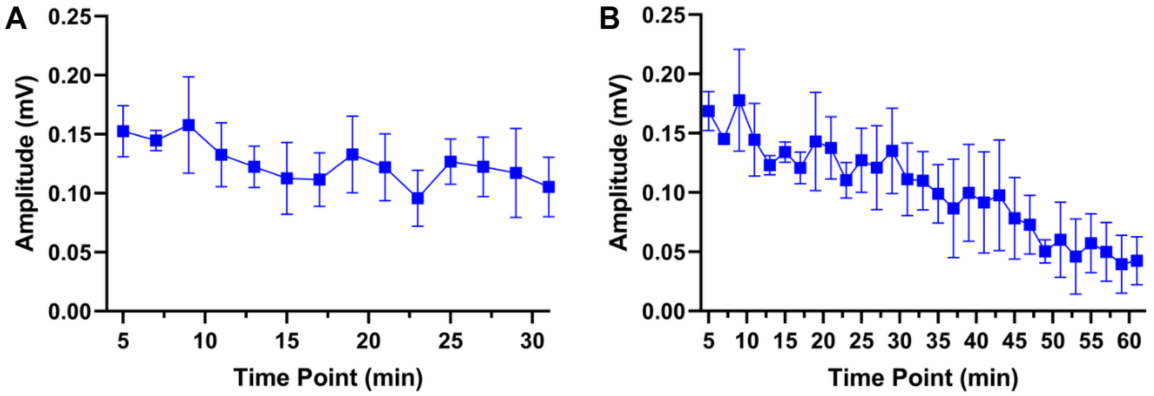

Figure SI1: Antenna Decay Following Excision. Negative peak amplitude (absolute value) as a response to 0.15g of coffee powder over time. **(A)** After 30 minutes of recording, showing 28.5% decay in intensity. N=5 antennae. **(B)** After 60 minutes of recording, showing 68.5% decay in intensity. N=3 antennae.

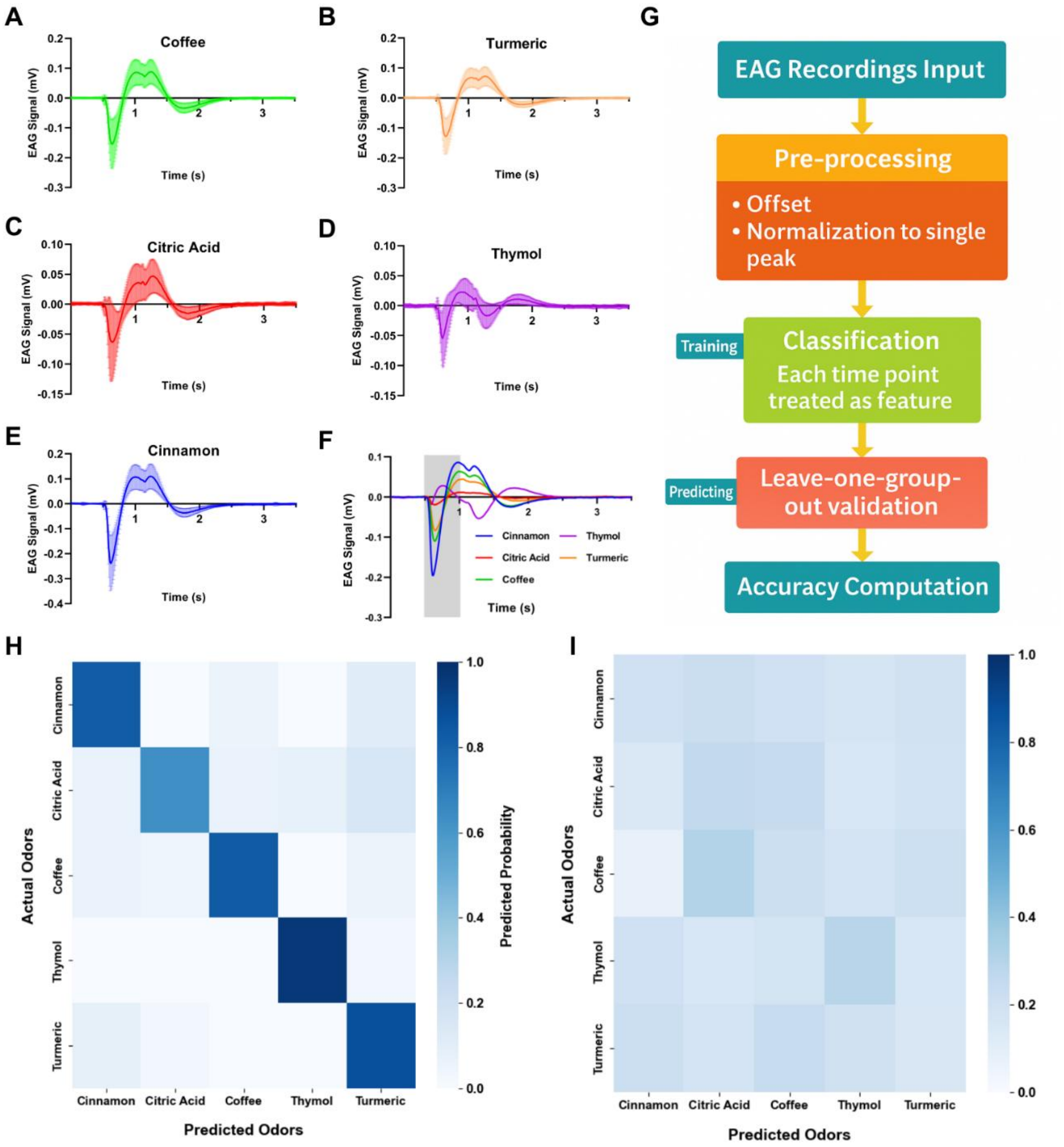

Figure. SI2: Solids Discrimination and Machine Learning Algorithm Training. (A-E) Averaged EAG signals as a response to 0.15g of different solids representing the training data set for each compound separately. Odorant name is indicated in the title of each graph. SD in light shades. N = 30 antennae, ~90 repetitions. **(F)** Averaged EAG signals of the same odorants after blank (response to clean air) reduction. The stimulus duration is represented by the gray square. N = 30 antennae, 445 repetitions. **(G)** Flow chart of the machine-learning algorithm used in this work. **(H)** Confusion matrix for the discrimination training dataset after blank subtraction using a random forest classifier, with an average accuracy of 82.7% over 445 samples. Two-tailed binomial test: $p < 0.0001$. **(I)** Control confusion matrix for panel (H) using randomized (shuffled) data with the random forest classifier, showing an average accuracy of 22.7% over 445 samples. Two-tailed binomial test: n.s.

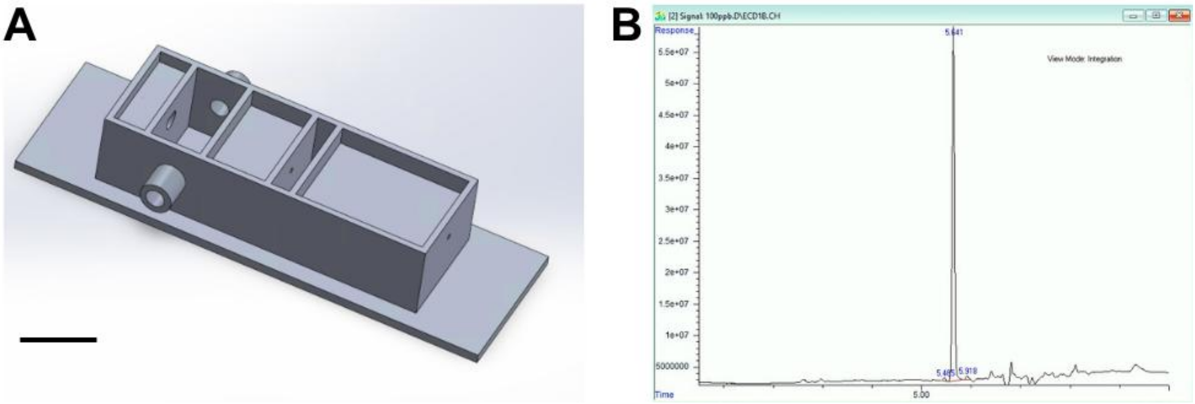

Figure SI3: GC-ECD Setup and Output Overview. (A) A SolidWorks® sketch of the specialized SPME fiber holder, designed to ensure similar exposure settings in both fiber-based and antenna-based experiments. scale bar= 1 cm **(B)** Example of GC-ECD output showing the response to TNT at a concentration of 100 ppb.

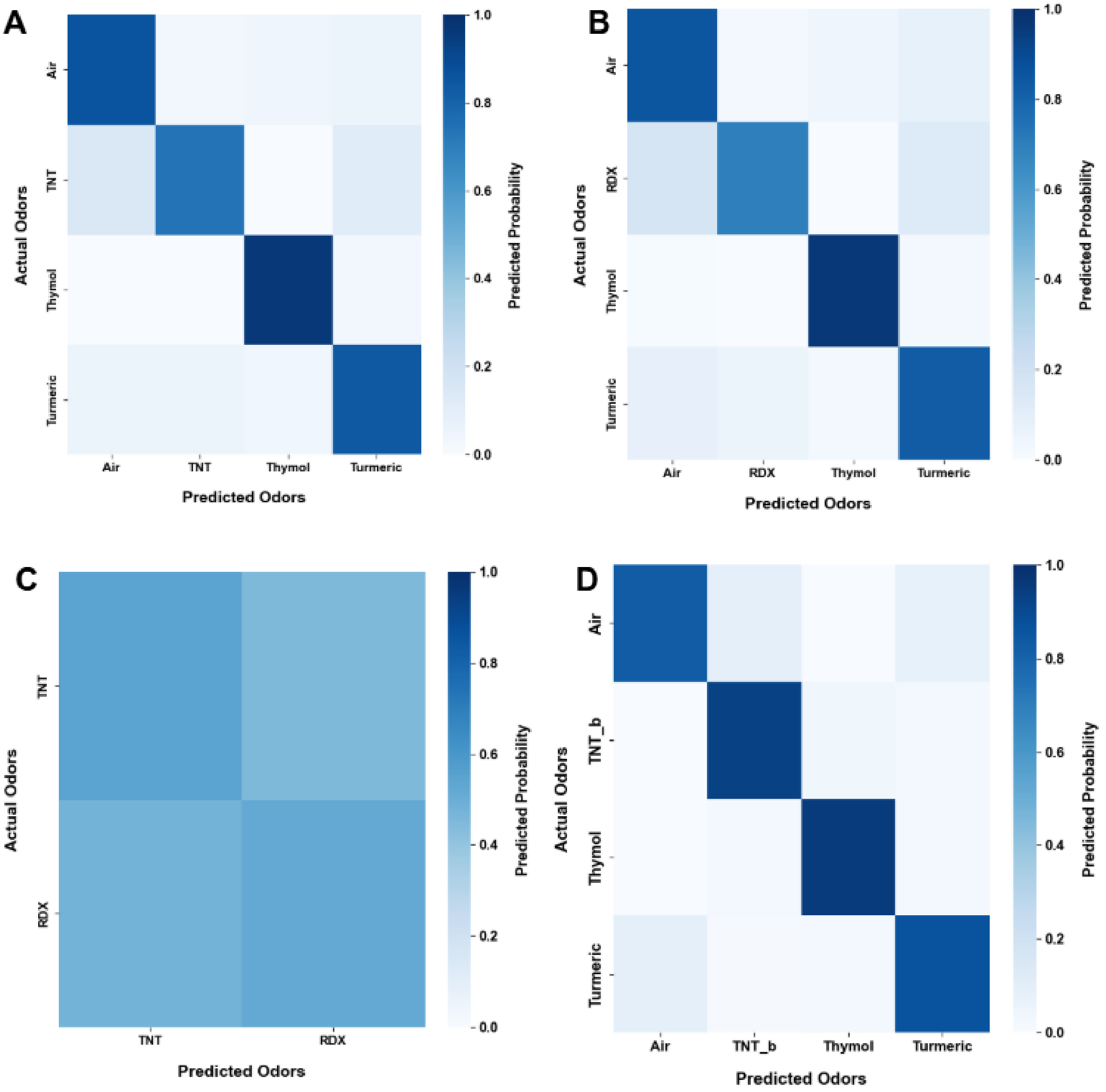

Figure SI4: Explosives Discrimination Test. (A) Confusion matrix for TNT vs. two other solids and air using a random forest classifier, with an average accuracy of 86.02% over 329 samples. Two-tailed binomial test: $p < 0.0001$. **(B)** Confusion matrix for RDX vs. two other solids and air using a random forest classifier, with an average accuracy of 84.5% over 329 samples. Two-tailed binomial test: $p < 0.0001$. **(C)** Confusion matrix for TNT vs. RDX using a random forest classifier, with an average accuracy of 53.62% over 138 samples. Two-tailed binomial test: n.s. **(D)** Confusion matrix for TNT with background odor (lemon) vs. two other solids and air using a random forest classifier, with an average accuracy of 89.55% over 335 samples. Two-tailed binomial test: $p < 0.0001$.

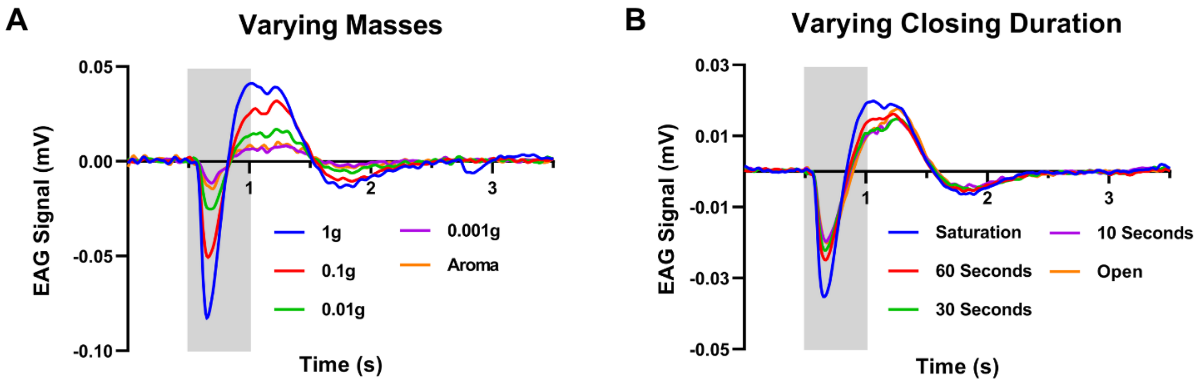

Figure SI5: Dose-Response Procedure Calibration. (A) Response profiles of the locust antenna to different masses of coffee powder ranging from 1g to 0.001g. Aroma indicates a vial emptied of its coffee but left unwashed. N = 7 locusts, 21 repetitions. **(B)** Response profiles of the locust antenna to different closing duration of an Eppendorf tube containing 1.5g of coffee powder. Saturation indicated 10 or more minutes of exposure. N = 6 locusts, 18 repetitions. In both, the stimulus duration is represented by the gray square.

References

1. Furan-2-ylmethanethiol | CAS#:98-02-2 | Chemsrc. https://www.chemsrc.com/en/cas/98-02-2_585984.html?utm_source=chatgpt.com#ebiemingDiv.
2. Cinnamaldehyde | C9H8O | CID 637511 - PubChem. https://pubchem.ncbi.nlm.nih.gov/compound/637511.
3. Hazardous Substances Data Bank (HSDB) : 2000 - PubChem. https://pubchem.ncbi.nlm.nih.gov/source/hsdb/2000.
4. Curcumin | C21H20O6 | CID 969516 - PubChem. https://pubchem.ncbi.nlm.nih.gov/compound/969516#section=National-Toxicology-Program-Studies.
5. Hazardous Substances Data Bank (HSDB) : 866 - PubChem. https://pubchem.ncbi.nlm.nih.gov/source/hsdb/866.
6. Ewing, R. G., Waltman, M. J., Atkinson, D. A., Grate, J. W. & Hotchkiss, P. J. The vapor pressures of explosives. *TrAC - Trends Anal. Chem.* **42**, 35–48 (2013).
7. Sulfur | S | CID 5362487 - PubChem. https://pubchem.ncbi.nlm.nih.gov/compound/5362487#section=Vapor-Pressure.
